# Vitamin D deficiency was common in all patients at a Swedish primary care centre, but more so in patients born outside of Europe

**DOI:** 10.1007/s10389-018-0910-z

**Published:** 2018-03-27

**Authors:** Per Wändell, Sahar Ayoob, Lennart Mossberg, Anna Andreasson

**Affiliations:** 1 Division of Family Medicine and Primary Care, Department NVS, Karolinska Institutet, Alfred Nobels allé 23, SE-141 83 Huddinge, Sweden; 2 Fornhöjden VC, Södertälje, Sweden; 3 Stress Research Institute, Stockholm University, Stockholm, Sweden

**Keywords:** Vitamin D, Immigrants, Gender

## Abstract

**Background:**

Vitamin D is associated with extra-skeletal processes, and vitamin D deficiency might contribute to the development of chronic diseases.

**Aim:**

To investigate vitamin D levels in an unselected patient population at a Swedish suburban primary care centre.

**Methods:**

Vitamin D levels were assessed in 102 patients aged 20 to 65 years visiting the primary care centre, independent of cause of visit, during 2 weeks in January 2014. The difference in vitamin D levels between patients born in Europe and patients born outside Europe was calculated using linear regression, adjusting for gender and age. The difference in prevalence of vitamin D deficiency (< 25 nmol/l) was calculated using logistic regression adjusting for gender, age, vitamin D supplement, and sun exposure.

**Results:**

Patients born outside Europe (*n* = 66) had 15 nmol/l [95% confidence interval (CI) 9.17–20.84] lower levels of vitamin D than patients born in Europe. Vitamin D deficiency was more common in patients born outside Europe (50%) than in patients born in Europe (11%, odds ratio 8.20 95% CI 2.49–26.98, *p* < 0.001).

**Conclusion:**

Lower levels of vitamin D and the prevalence of vitamin D deficiency were more common in patients born outside Europe compared to patients born in Europe.

## Introduction

The importance of vitamin D for a range of physiological processes has been highlighted in the last decade after the discovery of the extra-skeletal vitamin D receptor. The serum levels of vitamin D are the result of the amount of vitamin D that is absorbed from food and the amount that is produced in the skin under the influence of ultraviolet light from the sun, but deficiencies are very common worldwide (Holick and Chen 2008). Vitamin D deficiency might be a contributing cause for the increased risk for certain chronic diseases (Lamberg-Allardt et al. 2013), such as diabetes (Danescu et al. 2009), cardiovascular disease (Michaelsson et al. 2010), psychological diseases, immune dysfunction, and increased risk for colorectal-, prostate-, and lung cancer (Garland et al. 2009), and both low and high levels of vitamin D deficiency are linked to an increased mortality (Durup et al. 2015; Michaelsson et al. 2010).

Despite the higher latitude, the vitamin D status is better in the Nordic countries compared to southern European countries, to some extent due to a high consumption of fatty fish and vitamin D supplements such as cod liver oil in the Nordic countries (Lips 2007, 2010). Due to differences in skin tones, types of food consumed, and clothing, individuals that have immigrated from outside Europe to Sweden may have a higher risk of vitamin D deficiency than patients born in Europe. Considering the association between vitamin D deficiency and the risk for chronic disease, it is important to know the prevalence of vitamin D deficiency and insufficiency in primary care, and to identify risk groups for intervention.

The aim of this study was to assess the distribution of vitamin D levels and the prevalence of vitamin D deficiency in an unselected primary care population at a Swedish suburban primary care centre with a high proportion of patients who are born outside Europe, and to compare the prevalence of vitamin D deficiency between patients born outside of Europe and patients born in Europe.

## Methods

The primary care center is located in the town of Södertälje, a suburban area of Stockholm city, with a rather high rate of foreign-born individuals (38% in 2016), and also a low socio-economic status. The patient list of the primary health care center is 4.000 individuals, with 37% being foreign-born, and 33% born in Sweden with foreign-born parents. Out of the population of this town, 19% were classified as “open unemployed” in 2014, compared to 7.9% in the whole of Sweden. Two study physicians at the primary care center asked all their patients aged 20 to 65 years during 2 weeks in January 2014 to participate, independent of cause of visit, and 102 patients agreed. The total number of patients with GP appointments during these 2 weeks was approximately 320. Information on country of birth, age, sex, diabetes, sun exposure (recent travel), and vitamin D supplementation was collected at the primary care visit by the treating physician. Serum vitamin D (S-25(OH)D) levels were measured at the Karolinska University Hospital Laboratory. As cut-off for vitamin D deficiency and insufficiency, the values <25 nmol/l and < 50 nmol/l were used, respectively (Lamberg-Allardt et al. 2013).

Median values were used, with 95% confidence interval (95% CI), owing to skewed distribution. Linear regression (with b-coefficients and 95% CI) was used using vitamin D level as outcome, and logistic regression (with odds ratios, ORs, and 95% CI) using vitamin D deficiency as outcome. Two models were used in linear regression analysis. In model 1, country of birth, divided into European or non-European, was used as explanatory variables, and the model was adjusted for age, sex, self-reported vitamin D supplement and self-reported sun exposure. Model 2 included patients born outside of Europe only, and was adjusted for age, sex, years in Sweden, self-reported vitamin D supplement and self-reported sun exposure. Four models were used in the logistic regression analysis, with two models with all participants, model 1 country of birth, divided into European or non-European, was used as explanatory variables, and the model was adjusted for age and sex, and model 2 also for self-reported vitamin D supplement and self-reported sun exposure; and two models included patients born outside of Europe only, Model 3 adjusted for age, sex, and years in Sweden, and model 4 also self-reported vitamin D supplement and self-reported sun exposure. The study was approved by the Regional Ethical Board in Stockholm.

## Results

Sample characteristics are shown in Table 1. The five most common countries of birth were Irak (48%), Sweden (24%), Syria (6%), Finland (4%), and Turkey (4%). Patients born outside of Europe were somewhat younger, and had lower vitamin D levels than patients born in European. Linear regression (Table 2) showed a lower vitamin D level of 15 nmol/l in patients born outside Europe, and an effect of vitamin D supplement of 16 nmol/l in general, and of 22 nmol/l among non-European immigrants. Among patients born outside Europe, the longer the patient had lived in Sweden the higher the vitamin D levels (0.59 nmol/l per year). Patients born outside of Europe had an OR of 8 for vitamin D deficiency (<25 nmol/l) compared to patients born in Europe (Table 3). Categorizing patients as born in Sweden vs born outside Sweden, or Nordic vs non-Nordic country of birth, gave similar results (data not shown).

**Table 1:** Sample of patients with 25(OH) vitamin D measurements, by origin

	Born in Europe		Born outside Europe		Born in Europe	Born outside Europe	Difference for all born in or outside Europe
	Women (*n* = 26)	Men (*n* = 10)	Women (*n* = 49)	Men (*n* = 17)	Men and women (n = 36)	Men and women (n = 66)	*P*-value
Age (years)	54 (36.5–60)	51 (38.3–57)	37 (34–45.6)	45 (41–52)	53 (42.6–57)	41 (36–46.5)	0.027
25(OH) vitamin D-level	40 (38–54)	45 (21.6–54.7)	25 (20.1–31)	20 (16–26)	40 (38–52.8)	24 (19.5–28)	<0.001
Distribution of vitamin D-levels							<0.001
< 25 nmol/l	2 (7.7)	2 (20)	22 (44.9)	11 (64.7)	4 (11.1)	33 (50.0)	
25–49 nmol/l	14 (53.8)	3 (30)	21 (42.9)	5 (29.4)	17 (47.2)	26 (39.4)	
≥ 50 nmol/l	10 (38.5)	5 (50)	6 (12.2)	1 (5.9)	15 (41.7)	7 (10.6)	
Years in Sweden	–	–	7 (6–10.9)	10 (5–13)	–	7 (6–11)	–
Diabetes	1 (3.8)	2 (20)	3 (6.1)	0 (0)	3 (8.3)	3 (4.5)	0.66
Vitamin D supplement	4 (15.4)	1 (10)	10 (20.4)	0 (0)	5 (13.0)	10 (15.2)	0.86
Sun exposure	1 (3.8)	0 (0)	3 (6.1)	5 (29.4)	1 (2.8)	8 (12.1)	0.15

Median values (with 95% confidence intervals) with difference by Wilcoxon’s rank sum test for continuous variables, and numbers (%) for categorical variables with differences by Chi−square or Fisher’s exact test

**Table 2:** Linear regression models for vitamin D level (in nmol/l); model 1 includes all (*n* = 102), model 2 only non-European immigrants (*n* = 66)

Variable	Model 1	Model 2
	Coefficent (95% CI)	Coefficent (95% CI)
Age (per 1 year)	0.21 (−0.01; 0.44)	0.08 (−0.20; 0.37)
Sex (women)	4.44 (−1.85; 10.72)	3.60 (−3.79; 10.98)
Non-European immigrant	−15.00 (−20.84; −9.17)	–
Vitamin D supplement	15.98 (8.19; 23.77)	22.45 (13.64; 31.27)
Sun exposure	3.73 (−6.02; 13.48)	2.95 (−6.48; 12.39)
Years in Sweden	–	0.59 (0.19; 1.00)
Adjusted R-squared	0.37	0.40

**Table 3:** Logistic regression model for vitamin D deficiency (< 25 nmol/l)

	Vitamin D deficiency (< 25 nmol/l)			
	Model 1	Model 2	Model 3	Model 4
	OR (95% CI)	OR (95% CI)	OR (95% CI)	OR (95% CI)
Age (per 1 year)	0.97 (0.94–1.01)	0.98 (0.94–1.02)	0.99 (0.94–1.04)	1.00 (0.95–1.05)
Sex (women)	0.37 (0.13–1.05)	0.46 (0.16–1.35)	0.34 (0.09–1.22)	0.47 (0.12–1.78)
Non –European immigrant	8.05 (2.46–23.37)	8.20 (2.49–26.98)	–	–
Vitamin D supplement	–	0.23 (0.05–1.19)	–	0.22 (0.04–1.30)
Sun exposure	–	1.33 (0.29–6.17)	–	1.57 (0.31–7.85)
Years in Sweden	–	–	0.90 (0.82–0.98)	0.90 (0.82–0.98)
GoF	0.21	0.25	0.43	0.41

Models 1 and 2 includes all (*n* = 102), models 3 and 4 only non-European immigrants (*n* = 66)

## Discussion

The main finding of the study was the high rate of vitamin D deficiency and insufficiency among patients born outside of Europe compared to patients born in Europe, where half of the patients born outside of Europe had a vitamin D deficiency and only a tenth had adequate vitamin D levels.

The finding is consistent with a review of data from the Nordic countries presenting extremely low serum levels of vitamin D and a high prevalence of deficiency in immigrants from outside Europe and North America, specifically a very high prevalence of vitamin D deficiency among immigrant women from Arab countries, Pakistan, and Somalia, and among immigrant men from Pakistan (Wandell 2013). What our study adds is that non-European men showed lower vitamin D-levels and higher risk of vitamin D deficiency, which partly can be attributed to a lower intake of vitamin D supplements.

The primary health care center of the study is situated in an area with a high rate of foreign-born individuals (37% vs 14% in Sweden overall) and a rather low socio-economic status (the mean income is 90% of the Swedish mean), including a high unemployment rate, and the population in the catchment area could not be claimed to be representative for the Swedish population, but rather for suburbs with a high proportion of apartment buildings situated around the larger cities of Sweden, in this case Stockholm city. From a clinical point of view, the included patients did not differ from the main population of the patient list, although we have no exact data on this.

In this study, the levels of vitamin D were lower among men, in contrast to earlier reports (Wandell 2013). One possible explanatory factor could be that women seek care more often than men, who also tend to be more ill before seeking health care, a clinical experience from primary care. This pattern, with women having more symptoms and seeking care more often, while men have a shorter life expectancy and get their cardiac diseases around 5 years earlier than women (Forslund et al. 2014; Zarrinkoub et al. 2013), is referred to as the gender paradox, or the male–female mortality paradox (Oksuzyan et al. 2008). Otherwise, vitamin D deficiency is especially serious among pregnant women, since vitamin deficiency is associated with a higher risk of pregnancy and delivery complications (Grant et al. 2011), and also could affect fetal growth (Brunvand et al. 1996) and contribute to rickets among children (Elidrissy et al. 1984).

The study was performed during the winter, when the height of the sun in Sweden is too low to induce vitamin D production in the skin, and the serum levels of vitamin D depend on dietary intake and storage only. Future studies are needed to investigate if the difference in vitamin D levels and prevalence of vitamin D deficiency between patients born outside versus in Europe is present also during the summer.

Patients who reported taking vitamin D supplementation had higher levels of vitamin D than patients that did not, and of the 15 that took supplementation, only two had a vitamin D deficiency and half had adequate vitamin D levels. Although sun exposure was not found to be a significant factor for vitamin D levels in the present study, this may be due to the low number of exposed patients.

In a review of Turkish, Moroccan, Indian, and sub-Sahara African populations in Europe and their countries of origin, the vitamin D status was concluded to be low compared to indigenous European populations (van der Meer et al. 2011). A study comparing Tamil populations in Norway vs in Sri Lanka found a lower vitamin D level in Norway (Meyer et al. 2008). The most important factor for vitamin D in the body is through sun exposure, which is why higher vitamin D levels might be expected in sunnier countries, but in fact the levels do vary a lot (van der Meer et al. 2011), which could be influenced by sun exposure but also dietary intake. The dietary intake of vitamin D is higher in the Nordic countries than in other regions of the world (Lips 2007).

Limitations of the present study were the limited sample size, the lack of nutritional data, e.g., especially on intake of fish, lack of information with regard to educational level (Holvik et al. 2005), and lack of data concerning doses of vitamin D supplementation. The strengths included the fact that consecutive patients were included, during a limited time interval in the winter.

In conclusion, vitamin D deficiency and insufficient vitamin D levels were common at the primary care centre overall, and especially for patients born outside Europe.
